# A Personal Retrospective: Elevating Anandamide (AEA) by Targeting Fatty Acid Amide Hydrolase (FAAH) and the Fatty Acid Binding Proteins (FABPs)

**DOI:** 10.3389/fphar.2016.00370

**Published:** 2016-10-13

**Authors:** Dale G. Deutsch

**Affiliations:** Department of Biochemistry and Cell Biology, Stony Brook UniversityStony Brook, NY, USA

**Keywords:** anandamide transporter, fatty acid binding protein (FABP), fatty acid amide hydrolase (FAAH), anandamide synthesis, FAAH inhibitors, FABP inhibitors, anandamide, AEA

## Abstract

This perspective was adapted from a Career Achievement Award talk given at the International Cannabinoid Research Society Symposium in Bukovina, Poland on June 27, 2016. As a biochemist working in the neurosciences, I was always fascinated with neurotransmitter inactivation. In 1993 we identified an enzyme activity that breaks down anandamide. We called the enzyme anandamide amidase, now called FAAH. We and other laboratories developed FAAH inhibitors that were useful reagents that also proved to have beneficial physiological effects and until recently, new generations of inhibitors were in clinical trials. Nearly all neurotransmitters are water soluble and as such, require a transmembrane protein transporter to pass through the lipid membrane for inactivation inside the cell. However, using model systems, we and others have shown that this is unnecessary for anandamide, an uncharged hydrophobic molecule that readily diffuses across the cellular membrane. Interestingly, its uptake is driven by the concentration gradient resulting from its breakdown mainly by FAAH localized in the endoplasmic reticulum. We identified the FABPs as intracellular carriers that “solubilize” anandamide, transporting anandamide to FAAH. Compounds that bind to FABPs block AEA breakdown, raising its level. The cannabinoids (THC and CBD) also were discovered to bind FABPs and this may be one of the mechanisms by which CBD works in childhood epilepsy, raising anandamide levels. Targeting FABPs may be advantageous since they have some tissue specificity and do not require reactive serine hydrolase inhibitors, as does FAAH, with potential for off-target reactions. At the International Cannabis Research Society Symposium in 1992, Raphe Mechoulam revealed that his laboratory isolated an endogenous lipid molecule that binds to the CB1 receptor (cannabinoid receptor type 1) and this became the milestone paper published in December of that year describing anandamide (AEA, Devane et al., 1992). As to be expected, this discovery raised the issues of AEA's synthesis and breakdown.

## Anandamide synthesis

At first we mistakenly reported an enzymatic activity independent of the fatty acid amide hydrolase (FAAH) and calcium for the synthesis of AEA (Deutsch and Chin, 1993), but then followed up with collaborators to help elucidate the correct pathways. This misstep was caused by the condensation of ethanolamine with phenylmetylsulfony fluoride, whose product ran the same as AEA on thin layer chromatography (Bill Devane, personal communication circa 1994). The first demonstration of AEA synthesis via a calcium dependent N-acyl phosphatidylethanolamine-specific phospholipase D (NAPE-PLD) mechanism was reported in 1994 (Di Marzo et al., 1994) although this activity had been characterized with other phosphatidylethanolamines (Schmid et al., 1983). This enzyme was purified and cloned (Ueda et al., 2005) and subsequent papers using null mice confirmed that it was mainly responsible for the synthesis of AEA (Tsuboi et al., 2011; Leishman et al., 2016) although other minor pathways may be involved under certain conditions (Liu et al., 2008; Simon and Cravatt, 2010) depending upon the mouse construct (Leishman et al., 2016). FAAH can also mediate the reverse reaction for the synthesis of AEA and this has been implicated physiologically in liver regeneration (Devane and Axelrod, 1994; Arreaza et al., 1997; Izzo and Deutsch, 2011; Mukhopadhyay et al., 2011).

## Anandamide breakdown (anandamide amidase, FAAH)

**Figure 1:**
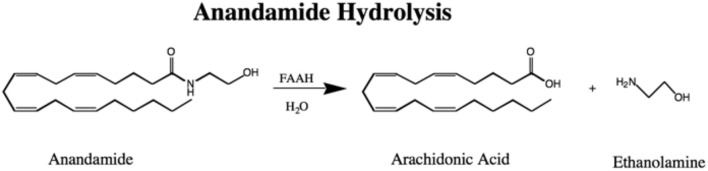
**The Hydrolysis of Anandamide to Arachidonic Acid and Ethanolamine by FAAH**.

In 1993 an enzyme we called anandamide amidase, now named called FAAH, was shown to break AEA down to arachidonic acid and ethanolamine (Figure 1) in the membrane fractions of most rat tissues except in leg and heart muscle (Deutsch and Chin, 1993). This activity was reported in liver microsomes for fatty acid amides, other than anandamide (Bachur and Udenfriend, 1966; Schmid et al., 1985). This lack of breakdown activity in muscle was fortuitous for the success of the vas deferens assay that was employed in the discovery of AEA in 1992 (Devane et al., 1992; Pertwee et al., 1995). In our original assay we used thin layer chromatography with AEA radio-labeled in the arachidonate portion of the molecule, but later ethanolamine labeled AEA simplified the assay procedure by permitting measurement of radiolabel without a thin layer chromatography step (Omeir et al., 1995). Cloning of the enzyme permitted more detailed molecular studies including ones that showed uniquely two serine residues in the active site (Omeir et al., 1999; Patricelli et al., 1999) and that FAAH was localized to the endoplasmic reticulum (Cravatt et al., 1996). FAAH is the main player in AEA inactivation although other pathways have been implicated in the metabolism of AEA as well (van der Stelt et al., 2002; Rahman et al., 2014).

## Early FAAH inhibitors

Phenylmethylsulfonyl fluoride (PMSF) was the first FAAH inhibitor, discovered serendipitously. When added to protect FAAH from proteolytic degradation in membrane fractions it had the opposite effect, completely inactivating the enzyme (Deutsch and Chin, 1993; Childers et al., 1994). PMSF was subsequently shown to raise AEA levels and have physiological activity and was surprisingly widely used in preclinical studies (70 PubMed references up to 2016) in spite of it being highly non-specific (Vann et al., 2012). The first systematic synthesis of FAAH inhibitors was undertaken at Stony Brook University in 1994 by Bohumir Koutek who made a series of fatty acid ethanolamides, α-keto ethanoamides, α-keto ethyl esters, and trifluoromethyl ketones, all reversible inhibitors. Arachidonyl trifluoromethyl ketone, the most specific, gave 100% inhibition at 7.5 μM (Ki = 650 nM) and Allyn Howlett, a co-author, found that it also bound to CB1 with only 21% occupancy at 10 μM. From studies with these transition state inhibitors, we knew that AEA was cleaved by a serine hydoxyl group on the enzyme. Realizing the clinical implications of raising AEA levels with inhibitors, the last sentence of our paper read: “The development of inhibitors that block the breakdown of anandamide may be significant therapeutically in any of the areas that Δ^9^-tetrahydrocannabinol and anandamide has been shown to play a role, including analgesia, mood, nausea, memory, appetite, sedation, locomotion, glaucoma, and immune function” (Koutek et al., 1994). Shortly thereafter, a series of fatty acid sulfonyl fluorides were synthesized with palmitylsulfonyl fluoride (AM374) being a 1000-fold more potent FAAH inhibitor than PMSF but did bind to CB1 (IC_50_ for AM374 was 520 nM using [^3^H]CP-55,940 in rat forebrain membranes, Deutsch et al., 1997a; Deutsch and Makriyannis, 1997). Also around this time, we and another group reported that methyl arachidonyl fluorophosphonate (MAFP) was a potent irreversible inhibitor (De Petrocellis et al., 1997; Deutsch et al., 1997b), an inhibitor that was later used for the crystallization of FAAH (Bracey et al., 2002). A series of MAFP analogs were synthesized and short chain saturated derivatives exhibited the highest *in vivo* potency (C8:0 and C12:0, Martin et al., 2000). Around this time the first report of NSAIDs inhibiting FAAH was published as well as a review covering other inhibitors (Fowler et al., 1997; Boger et al., 1999; Ueda et al., 2000).

## Later FAAH inhibitors and clinical trials

The “golden age,” with hundreds of FAAH inhibitors developed, followed these early reports, in part as a result of cloning, crystal structure determination, and the development of assays (activity-based protein profiling to determine off-target reactions, Cravatt et al., 1996; Leung et al., 2003; McKinney and Cravatt, 2005; Mileni et al., 2008; Fowler, 2015). These inhibitors compromise, for example; α-ketoheterocycle, carbamate (e.g., URB597), aryl and piperidine/piperazine ureas (e.g., PF-3845, PF-04457845), azetidine urea, azetidine, boronic acid, azole, and ethylaminopyrimidines, and tetrahydronaphthyridine, derivatives from a variety of academic and industrial institutions (Khanna and Alexander, 2011; Fowler, 2015; Lodola et al., 2015). Recently, Phase 1 clinical trials with BIA 10-2474 (from the Bial pharmaceutical company) were terminated owing to the death and sickening of some volunteers (Casassus, 2016). However, a Phase 2 trial with the Pfizer FAAH inhibitor PF-04457845 was completed and the compound was well tolerated in osteoarthritis patients but there was a lack of analgesic effect in the knee (Huggins et al., 2012). Johnson and Johnson suspended its Phase 2 clinical trial with JNJ-42165279 for anxiety and depression owing to the Bial results, as did Pfizer with PF-04457845 for Tourette Syndrome and cannabis dependence trials.

## Cellular uptake of anandamide is coupled to its breakdown by FAAH

**Figure 2:**
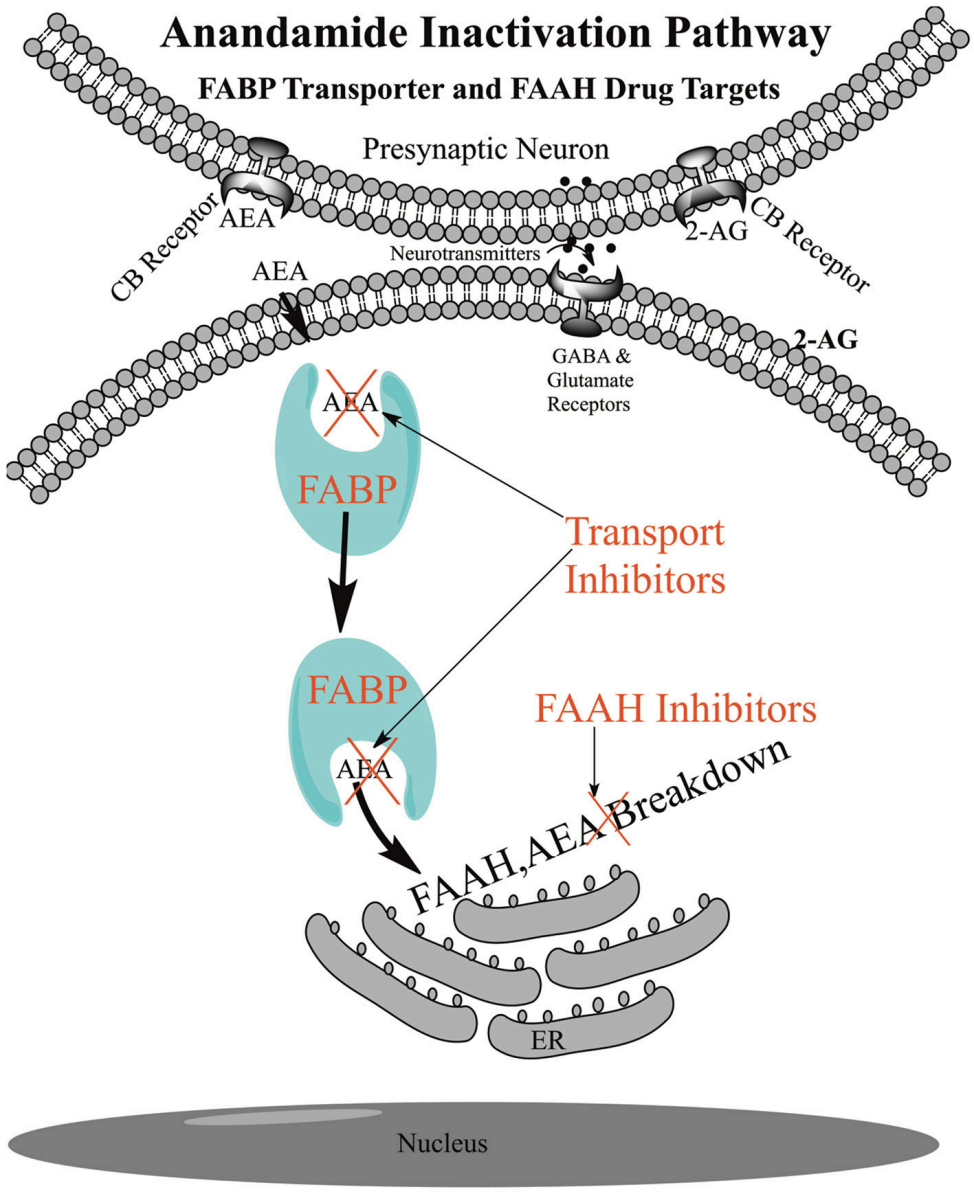
**Schematic of Anandamide Uptake and Inactivation**. Endogenous anandamide (AEA) passes through the cellular membrane without the need for a protein transporter and is shuttled through the aqueous environment of the cytoplasm with the fatty acid binding protein transporters (FABPs) to endoplasmic reticulum (ER) localized fatty acid amide hydrolase (FAAH) for catabolism. FAAH drives the uptake and inhibition of FAAH or the FABPs reduces the rate of anandamide breakdown and raises the AEA levels for signaling at the receptor.

The uptake of AEA into cells is unique in that its uptake is coupled to its breakdown by the catabolic enzyme FAAH located at the endoplasmic reticulum (Figure 2). Uptake rates in different cells are generally correlated with inherent FAAH concentration and the rate increases further with transfection of FAAH (Day et al., 2001; Deutsch et al., 2001). Conversely, uptake rates are generally negatively correlated with the degree of FAAH inhibition, although in some cells other catabolic enzymes and their inhibitors may play a role, such as COX-2 and NAAA (Fowler et al., 2004, 2013; Glaser et al., 2005; Hillard and Jarrahian, 2005). FAAH removes AEA from inside the cell, disrupting the equilibrium between inside and outside the cell, generating a concentration gradient that drives uptake (Figure 2).

## Transmembrane transporters

In 1993 we were the first to show, with rather rudimentary experiments, that AEA was actively taken up in neuroblastoma and glioma cells (Deutsch and Chin, 1993). In 1994 the uptake of AEA was confirmed and the mechanism was postulated to involve an ATP independent active membrane transporter (Di Marzo et al., 1994). The hypothesis of an AEA transmembrane transporter became dogma for many years and the “hunt” still goes on for this “putative” anandamide membrane transporter (AMT) also called the “putative endocannabinoid membrane transporter (EMT, Ligresti et al., 2010; Nicolussi et al., 2014; Nicolussi and Gertsch, 2015). Many of the AMT (EMT) proposals have fallen by the wayside. For example, a paper first showed uptake was FAAH independent and then a decade later it was proposed that a FAAH fragment called FLAT (FAAH-like anandamide transporter) was the transmembrane transporter (Fegley et al., 2004; Fu et al., 2012), the latter being questioned (Leung et al., 2013; Björklund et al., 2014; Fowler, 2014). The evidence for a transmembrane transporter was based on enzyme saturation kinetics in cell culture, uptake studies in cells and the physiological effects of “membrane transporter inhibitors.” Many dozens of such inhibitors were proposed. However, it was shown that the kinetics of uptake of AEA can show saturation owing to the passage of hydrophobic AEA through the water layer surrounding the cell and that many of these transport inhibitors were in fact FAAH inhibitors or FAAH substrates or bound to receptors confounding the mechanism of their physiological effects (Glaser et al., 2003; Alexander and Cravatt, 2006; Bojesen and Hansen, 2006; Nicolussi and Gertsch, 2015). Furthermore, it was demonstrated that AEA can freely pass through an artificial membrane without the aid of any protein (Figure 2, Bojesen and Hansen, 2005; Di Pasquale et al., 2009; Kaczocha et al., 2012a; Fowler, 2013, 2015). A transmembrane protein transporter has not been identified to date and the effects of these inhibitors appear to occur downstream and many of the so-called transporter inhibitors were in fact FAAH or FABP inhibitors.

## FABPs: intracellular transporters for anandamide

FABPs are “workhorse” proteins for shuttling lipids inside the cell (Furuhashi and Hotamisligil, 2008). From the observation that cultured cells accumulate AEA in excess of that found in the media, we and others postulated that cells may have an intracellular binding protein(s) (Hillard and Jarrahian, 2000; Rakhshan et al., 2000; Deutsch et al., 2001). In 2009, FABPs were identified by us to be intracellular carriers for AEA (Figure 2). Our conclusion was based upon the observation that AEA uptake and hydrolysis were significantly potentiated in N18TG2 neuroblastoma cells after overexpression of FABP5 or FABP7 or in COS-7 cells stably expressing FAAH. Administration of the competitive FABP ligand oleic acid or the non-fatty acid FABP inhibitor BMS309403 attenuated AEA uptake and hydrolysis confirming the roles of FABP as AEA carriers (Kaczocha et al., 2009). Shortly thereafter, molecular dynamics simulations of AEA in complex with FABP7 showed that the carboxamide oxygen of AEA can interact with FABP7 interior residues R126 and Y128, while the hydroxyl group of AEA can interact with FABP7 interior residues, T53 and R106 (Howlett et al., 2011). Using more detailed structural crystallographic studies we determined that AEA (as well as 2-AG) bound to key amino acid residues consistent with that observed for fatty acids and the corresponding polar groups for the endocannabinoids (Sanson et al., 2014).

## Inhibitors of the FABPs and binding of cannabinoids

Specific inhibitors of the FABPs were developed at Stony Brook such as SBFI26 that led to an increase in AEA levels in the brains of animals and had physiological effects. As shown in Figure 2, inhibiting the FABPs will reduce the AEA delivery to FAAH and disrupt the outward/inward concentration gradient driven by FAAH. Intriguingly, the truxillic acid structure of SBFI26 is the core structure of (−)-incarvillateine, the active component from a Chinese herb used for rheumatism (Berger et al., 2012). It was found that some of the inhibitors (such as OMDM1, OMDM2, VDM11, AM1172, AM404) of the “putative” transmembrane transporter, inhibit FABPs, perhaps explaining, in part, their mechanism of action (Kaczocha et al., 2012b).

Recently, again using computational analysis and ligand displacement assays, we showed that human FABP3, 5, and 7 bind THC and CBD and function as intracellular carriers (Elmes et al., 2015). Furthermore, we demonstrated that THC and CBD inhibit the cellular uptake and catabolism of AEA by targeting FABPs. This competition for FABPs may in part or wholly explain the increased circulating levels of AEA reported after consumption of cannabidiol (Leweke et al., 2012). These data may explain, in part, the action of CBD in modulating the endocannabinoid tone *in vivo* and its reported efficacy toward epilepsy and other neurological disorders (Ibeas Bih et al., 2015). The role of the FABP as carriers for endocannabinoids and particularly AEA will undoubtedly grow as illustrated in the liver where FABP1 also serves as an AEA carrier (Schroeder et al., 2016).

## The future

Inhibition of FAAH or FABPs decrease the breakdown of AEA leading to less cellular uptake and prolonged physiological effects. The Bial clinical trial has temporarily set back the approach of employing a FAAH inhibitor. However, other FAAH inhibitors have been shown to be safe in Phase 2 clinical studies and these may be pursued in the future for indications, for example, such stress-related disorders. FABP inhibitors provide another approach for raising AEA levels. Since FABPs have some tissue specificity, it may be possible to design inhibitors that target specific organs, such as the brain, more easily than with FAAH inhibitors.

## Author contributions

The author confirms being the sole contributor of this work and approved it for publication.

## Funding

The work of my laboratory had been generously funded by the National Institute on Drug Abuse, intermittently, since the early 1980s. These grants funded the work for the discovery of FAAH, the study of its inhibitors, the identification of the FABPs as AEA carriers and most recently, the drug discovery program for FABP inhibitors (NIH 035923). Dr. Hillery, Rapaka and Volkow have always been generous with their advice over the years.

### Conflict of interest statement

The author declares that the research was conducted in the absence of any commercial or financial relationships that could be construed as a potential conflict of interest.

## Abbreviations

NAPE-PLD: N-acyl phosphatidylethanolamine-specific phospholipase D
NSAID: Nonsteroidal anti-inflammatory drug
AEA: anandamide
FAAH: fatty acid amide hydrolase
FABP: fatty acid binding protein
ER: endoplasmic reticulum
CB receptor: cannabinoid receptor
COX-2: cyclooxygenase 2
NAAA: N-acylethanolamine-hydrolyzing acid amidase
2-AG: 2-arachidonylglycero
tetrahydrocannabinol: THC
cannabidiol: CBD.
